# Relationship between serum trough levels and efficacy of brodalumab from a *post hoc* exploratory analysis of a Japanese study in patients with plaque psoriasis

**DOI:** 10.1111/1346-8138.15690

**Published:** 2020-11-08

**Authors:** Yukie Yamaguchi, Yasumasa Kanai, Hiroki Kitabayashi, Hiroki Okada, Hidemi Nakagawa

**Affiliations:** ^1^ Department of Environmental Immuno‐Dermatology Yokohama City University Graduate School of Medicine Yokohama Japan; ^2^ Medical Affairs Kyowa Kirin Co., Ltd. Tokyo Japan; ^3^ Clinical Sciences Research Laboratories Translational Research Unit, R&D Division Kyowa Kirin Co., Ltd. Tokyo Japan; ^4^ Department of Dermatology The Jikei University School of Medicine Tokyo Japan

**Keywords:** biologics, brodalumab, interleukin‐17 receptor A, pharmacodynamics, plaque psoriasis

## Abstract

Previous clinical studies have shown that efficacy and serum brodalumab levels are dose dependent in patients with psoriasis receiving the same dose of brodalumab during the study. This study aimed to investigate the association between dosage, serum levels, and efficacy of brodalumab in Japanese patients with plaque psoriasis with dosage variations during the study. This was a *post hoc* exploratory analysis of a 108‐week, multicenter, open‐label extension study, which changed into a post‐marketing surveillance study following brodalumab approval in Japan. Eligible patients with plaque psoriasis (*n* = 129) received brodalumab 140 mg every 4 weeks on Day 1; dosage change at physician’s discretion from 140 mg every 8 weeks to 210 mg every 2 weeks was permitted; patients switched to 210 mg every 2 weeks during the post‐marketing surveillance study. Exploratory endpoints included serum brodalumab levels at Weeks 28 and 108, its association with Psoriasis Area and Severity Index score, and Psoriasis Area and Severity Index score in patients receiving brodalumab 210 mg every 2 weeks at end of study. Median brodalumab trough levels were significantly higher (*P* < 0.05) at higher vs. lower dosages at Weeks 28 (*n* = 126) and 108 (*n* = 111) except for 140 mg every 2 weeks vs. 210 mg every 2 weeks at Week 108 and higher in patients with lower Psoriasis Area and Severity Index scores—significantly different only for Psoriasis Area and Severity Index score 0 vs. >2 at Week 28 (*P* = 0.0153). Of 100 patients receiving 210 mg every 2 weeks at end of study, 89% had a Psoriasis Area and Severity Index score ≤2. In patients with plaque psoriasis, brodalumab efficacy may depend upon sustained serum trough levels and can be restored by using the approved dose.

## Introduction

Psoriasis, a chronic systemic inflammatory disorder, is estimated to affect more than 125 million people globally.^1^ Plaque psoriasis, the most common phenotype, is characterized by erythematous, indurated, scaly plaques on the scalp, trunk, and extremities,^1^ and is reported in 55–60% and 97.4% of patients with psoriasis in the United States (US)^2^ and Japan in 2016 and 2015,^3^ respectively.

Guidelines recommend biologic treatment in patients with plaque psoriasis with inadequate/no response to standard systemic therapies or phototherapy.^4^, ^5^ Brodalumab, a fully human anti‐interleukin (IL)‐17 receptor A monoclonal antibody, is approved for the treatment of plaque psoriasis in several countries, including Japan,^6^ USA^7^ and in the European Union.^8^ The efficacy and safety of brodalumab in patients with moderate‐to‐severe plaque psoriasis were demonstrated in both global^9^, ^10^, ^11^ and Japanese^12^, ^13^ studies. In the Japanese Phase II study, a dose‐dependent increase in mean percentage improvement in Psoriasis Area and Severity Index (PASI) scores (70 mg/140 mg/210 mg: 37.7%/82.2%/96.8%) and response rates (PASI 75/90/100: 70 mg, 25.6%/15.4%/2.6%; 140 mg, 78.4%/64.9%/35.1%; 210 mg, 94.6%/91.9%/59.5%) at Week 12 was observed with brodalumab.^12^

Population pharmacokinetic model‐based studies using data from healthy individuals and/or patients with psoriasis have shown that body weight impacts the volume of distribution and clearance of brodalumab.^14^, ^15^ In the Japanese Phase II study, the PASI response rate decreased with increasing body weight with brodalumab 140 mg but was not substantially impacted at 210 mg.^12^ Further, in patients with psoriasis receiving ustekinumab, an anti‐IL‐12/23 p40, the PASI 75 response rate was lower in those with body weight >100 kg at lower vs. higher doses.^16^ However, dosing did not impact the response rate in those with body weight ≤100 kg, suggesting that serum drug levels were influenced by body weight, which in turn impacted efficacy. Similarly, in an exposure–response modeling study, higher serum levels of ixekizumab, an anti‐IL‐17A, was associated with higher PASI response rate, and higher body weight was associated with lower serum trough levels in patients with moderate‐to‐severe psoriasis.^17^

Thus, previous studies in patients with psoriasis receiving biologics suggest an association between dose, serum drug levels, and efficacy. However, all these studies compared patients using the same dose over the study period. Therefore, this *post hoc* exploratory analysis of a long‐term study investigated the precise association between efficacy and serum brodalumab levels in patients with plaque psoriasis with dosage variations.

## Methods

### Study design

This was a *post hoc* exploratory analysis of a multicenter, open‐label, long‐term extension study (NCT02052609)^18^ in patients with plaque psoriasis (psoriasis vulgaris, psoriatic arthritis), which changed into a post‐marketing surveillance (PMS; NCT04183881; July 04 to December 31, 2016)^18^ study following approval of brodalumab 210 mg every 2 weeks (Q2W) in Japan.

### Patient criteria

Patients with plaque psoriasis who transitioned from a Phase III study of brodalumab in Japan^13^ were included in this analysis. Detailed patient criteria have been previously reported.^18^

### Treatment

**Figure 1:**
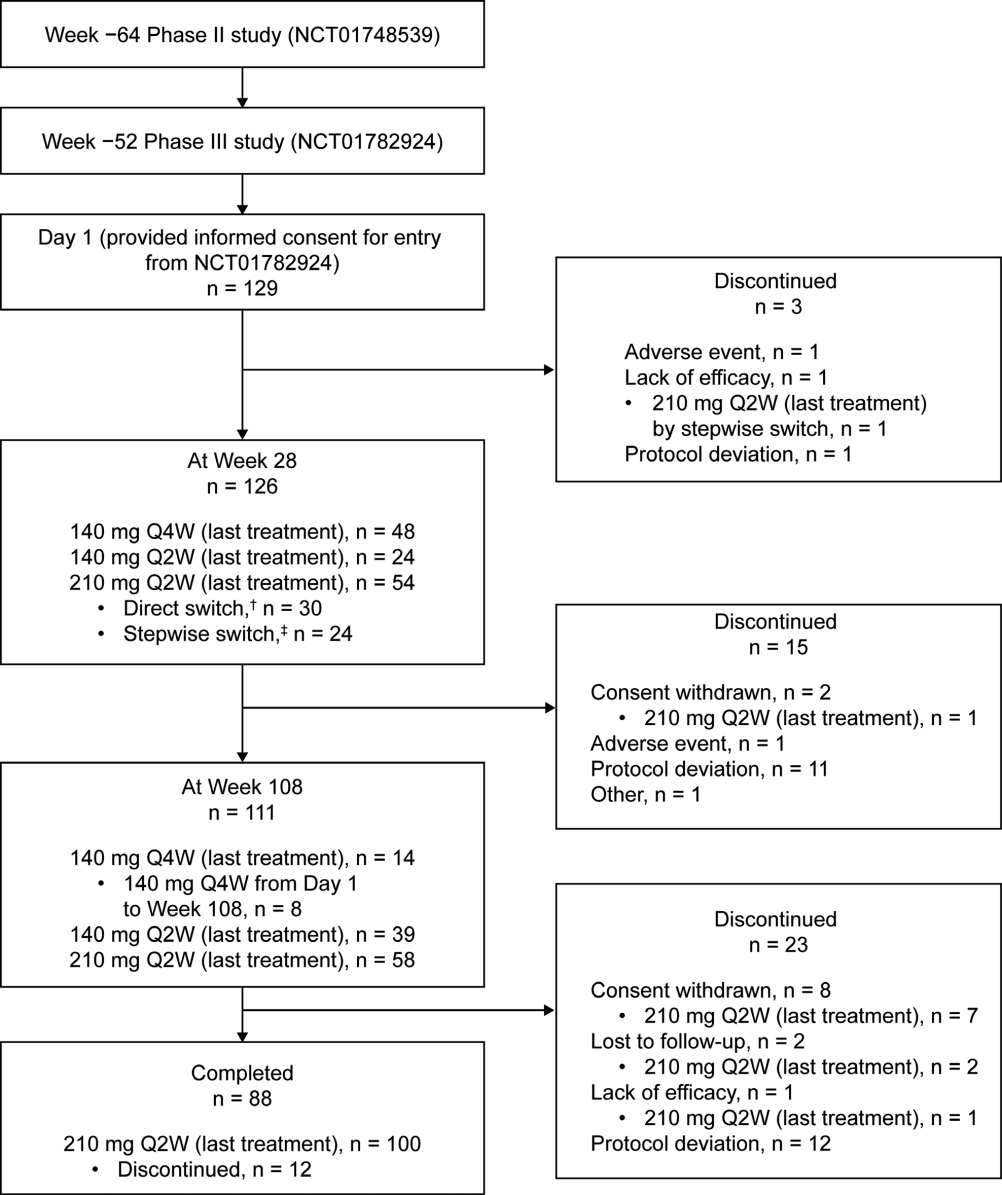
Patient disposition. Q2W, every 2 weeks; Q4W, every 4 weeks. ^a^Dose was directly escalated from 140 mg Q4W to 210 mg Q2W. ^b^Dose was escalated in a stepwise manner (140 mg Q4W → 140 mg Q2W → 210 mg Q2W).

In the preceding Phase III study (Fig. 1), patients received subcutaneous brodalumab 140 mg every 4 weeks (Q4W) until the end of study (EOS; Day 1 of the current study).^18^ From Week 2 to Week 28, the dosage could be maintained at 140 mg Q4W or switched directly to 210 mg Q2W, the dose could be maintained and dosing interval shortened (140 mg Q4W → 140 mg Q2W), or dose could be escalated and dosing interval shortened in a stepwise manner (140 mg Q4W → 140 mg Q2W → 210 mg Q2W) at the physician’s discretion based on the static Physician’s Global Assessment (sPGA) score (≥3 or maintained at 2 [mild] for ≥4 weeks on a 6‐point scale: 0, clear/no apparent disease; to 5, severe disease).^10^ From Day 1 to Week 28, sPGA was measured bi‐weekly. Following Week 28 until the start of the PMS, besides dose escalations and changes in dosing frequency (140 mg Q2W/Q4W/every 8 weeks [Q8W] or 210 mg Q2W), stepwise dose reductions were also permitted at the physician’s discretion. All patients were switched to the approved brodalumab dose at the start of the PMS study.^18^

The study protocol was approved by the institutional review board at each study site. The study was performed in compliance with the Declaration of Helsinki and Good Clinical Practice and Good Post‐marketing Study Practice guidelines. All patients provided written informed consent.

### Endpoints and assessments

Exploratory efficacy endpoints for the *post hoc* analysis included serum brodalumab levels and its association with PASI score at Weeks 28 and 108 and PASI score in patients receiving brodalumab 210 mg Q2W at EOS (date of study completion or discontinuation). Serum brodalumab levels were assessed at Weeks 12 and 28 and every 16 weeks thereafter until EOS. PASI score was assessed 4‐weekly until Week 28 and 8‐weekly thereafter until EOS.

Other assessments included change in serum brodalumab levels and PASI score in patients who met the sPGA score criteria after Day 1 and switched to 210 mg Q2W by Week 28. Among patients receiving 210 mg Q2W at EOS, the following were evaluated: serum brodalumab levels and PASI score by body weight (<55 kg, ≥55–<70 kg, ≥70–<85 kg, ≥85 kg), and serum brodalumab levels and PASI score in non‐responders at Day 1. In this study, severity of psoriatic lesions was measured using both PASI score and PASI achievement rate. Although PASI 90 is defined as a treatment goal in randomized clinical trials, a PASI score of 2 as a treatment goal^19^ in daily clinical practice can provide a potentially better assessment of psoriasis severity when the PASI achievement rate cannot be calculated due to the absence of baseline values.^20^ PASI improvement was calculated as percentage improvement from baseline and was negatively expressed for increased scores from baseline; failure to achieve ≥75% improvement in PASI score from baseline (PASI 75) was considered as no response.

### Serum brodalumab level measurement

Blood samples were collected at the clinical trial sites by SRL Medisearch, Inc. (Shinjuku‐ku, Tokyo, Japan); serum brodalumab levels were measured centrally at TANDEM labs (West Trenton, NJ, USA). Following blood sample centrifugation, serum was frozen at ≤−20°C and brodalumab levels were quantified by an enzyme‐linked immunosorbent assay (ELISA; lower limit of quantification [LLOQ] = 0.05 µg/mL; below LLOQ = 0) using murine anti‐brodalumab antibodies—capture antibody and a horseradish peroxidase antibody. The ELISA was developed with 3,3′,5,5′‐tetramethylbenzidine and hydrogen peroxide, and optical density signals were measured at 450 and 650 nm. Brodalumab levels were determined based on a four‐parameter regression calibration curve generated using optical density signal measurements.

### Statistical analysis

This was a *post hoc* exploratory analysis of a non‐randomized study. Serum brodalumab trough level and PASI score were analyzed for all dosage groups (140 mg Q4W, 140 mg Q2W, and 210 mg Q2W) at Weeks 28 and 108 (additional timepoint, close to the approval date, was included for analysis). The difference in serum brodalumab levels by dosage and PASI score was tested using the Kruskal–Wallis test and confirmed by the Steel–Dwass test in case the difference was significant. Serum brodalumab trough levels for patients with a PASI score >2 at each timepoint were further classified by PASI score (2.1–5 or >5). The correlation between serum brodalumab levels and PASI score at Weeks 28 and 108 was analyzed using the Spearman’s rank‐correlation coefficient.

The PASI scores were summarized for baseline, Day 1, switching point, and Week 28 for patients switching from 140 mg Q4W to 210 mg Q2W after loss of efficacy; if discontinuation occurred before Week 28, the discontinuation timepoint was considered as Week 28. PASI scores at Day 1 and Week 28 were compared with that at the switching point as control using the Steel–Dwass test accounting for multiplicity. For patients who maintained 140 mg Q4W from Day 1 to Week 108, PASI score was summarized for each measurement timepoint.

To assess the effect of body weight on the relationship between serum brodalumab levels and PASI score, patients receiving 210 mg Q2W at EOS were categorized by body weight, and PASI score and serum brodalumab levels were presented for each group. Additionally, a multiple linear regression model was applied to explore an association with serum brodalumab trough level and body weight as covariates and PASI score as the outcome; all variables were considered as continuous values. Serum brodalumab trough level and PASI response were summarized at each measurement timepoint for patients receiving 210 mg Q2W at EOS and not achieving PASI 75. In these patients, body weight was also summarized at each measurement timepoint for those with a poor response on Day 1 of the study.

To ensure accurate estimation of brodalumab trough levels, a missed dose beyond ± 7 days of the specified assessment day was included as a protocol deviation for this analysis, unlike for the safety analysis.^18^ For any unscheduled change in dosage, data at the next visit were considered representative for the switching point.

Data were summarized using median (interquartile range [IQR]). Outliers were excluded from the graphical data presentation but included in the analyses. Statistical analyses were conducted using a two‐sided significance level of 0.05. All analyses were performed using SAS software, version 9.2 or 9.3 (SAS Institute, Inc., Cary, NC, USA).

## Results

### Patient disposition and baseline characteristics

Of the 129 enrolled patients, three discontinued, one each because of adverse event, lack of efficacy, and protocol deviation (Fig. 1). Between Weeks 2 and 28, 55 patients switched from 140 mg Q4W to 210 mg Q2W; 54 patients had 210 mg Q2W as the last treatment dose at Week 28 and one patient who underwent a stepwise switch discontinued treatment before Week 28 due to lack of efficacy. Of the remaining 126 patients at Week 28, 111 completed 108 weeks and 15 discontinued. Overall, 88 patients completed the study. Of the 129 patients, 80.6% were male, and the median (IQR) characteristics at baseline/Day 1 were as follows: age, 43.0 (37.0–55.0) years; body weight, 72.0 (62.5–84.5) kg/69.5 (60.9–83.8) kg; PASI score, 24.7 (18.0–32.4)/0.00 (0.00–1.20); and DLQI score, 8.5 (5.0–15.0)/1.0 (0.0–2.0).^18^

### Treatment with brodalumab

The initial dosage of 140 mg Q4W was maintained in 48 patients up to Week 28 and in 14 patients up to Week 108 (Fig. 1). Nine patients switched their dosage at Week 2. At EOS, 100 patients were receiving 210 mg Q2W.

### Serum brodalumab levels

**Figure 2:**
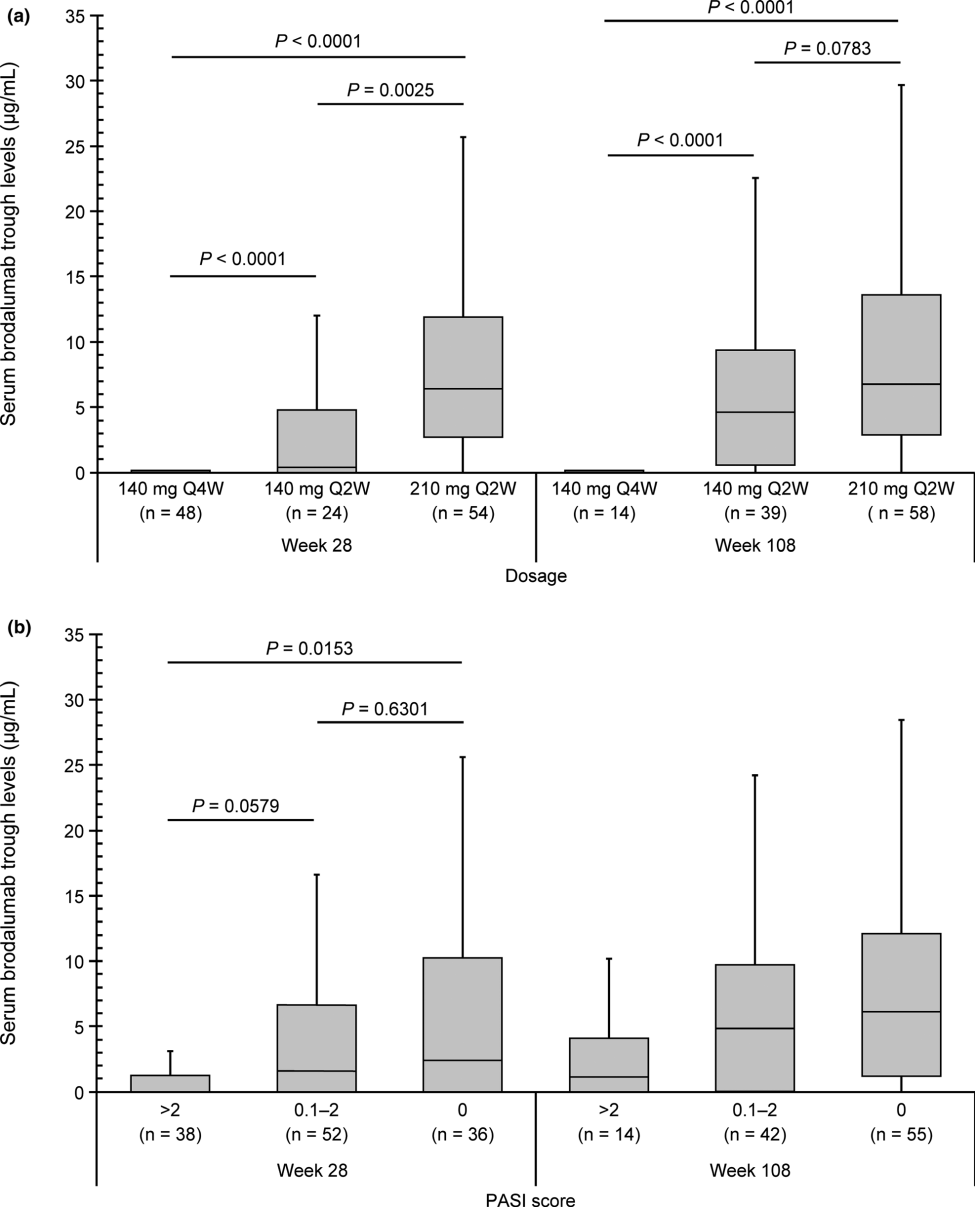
Serum brodalumab levels at Weeks 28 and 108. (a) By dosage^a^. (b) By PASI score^b^. Q2W, every 2 weeks; Q4W, every 4 weeks; PASI, Psoriasis Area and Severity Index. The horizontal lines in each box represent the following values: top, quartile 3; middle, median; bottom, quartile 1. The vertical lines on each box represent: top: quartile 3 + 1.5 × interquartile range; bottom: minimum values. ^a^ *P*‐value between the three dosage groups by the Kruskal–Wallis test was <0.0001 at Weeks 28 and 108. *P*‐values between the three dosage groups by the Steel–Dwass test at Week 28 were <0.0001 (140 mg Q4W vs. 210 mg Q2W, 140 mg Q4W vs. 140 mg Q2W) and 0.0025 (140 mg Q2W vs. 210 mg Q2W) at Week 28 and <0.0001 (140 mg Q4W vs. 210 mg Q2W, 140 mg Q4W vs. 140 mg Q2W) and 0.0783 (140 mg Q2W vs. 210 mg Q2W) at Week 108. ^b^ *P*‐value between the three PASI score groups by the Kruskal–Wallis test was 0.0135 and 0.0836 at Week 28 and Week 108, respectively. *P*‐value between the three PASI score groups by the Steel–Dwass test were 0.0153 (140 mg Q4W vs. 210 mg Q2W), 0.0579 (140 mg Q4W vs. 140 mg Q2W), and 0.6301 (140 mg Q2W vs. 210 mg Q2W) at Week 28.

Median brodalumab trough levels were 0 μg/mL with 140 mg Q4W at Weeks 28 and 108 but increased with increasing dosage at both timepoints. Trough levels were significantly higher at higher vs. lower dosages at both timepoints except for 140 mg Q2W vs. 210 mg Q2W at Week 108 (Fig. 2a).

Similarly, median brodalumab trough levels were higher in patients with lower PASI scores (Fig. 2b); however, the difference was significant only for PASI score >2 vs. 0 at Week 28 (*P* = 0.0153). Further, a significant weak correlation was found between serum brodalumab levels and PASI score at Weeks 28 (ρ = −0.2713, *P* = 0.0021) and 108 (ρ = −0.2322, *P* = 0.0142). Upon further stratification of patients with PASI score >2 into 2.1–5 and >5, median trough levels were 0 μg/mL in patients with PASI score >5 at Weeks 28 and 108 (Fig. S1).

### Efficacy in patients on 210 mg Q2W at EOS and Week 28

**Figure 3:**
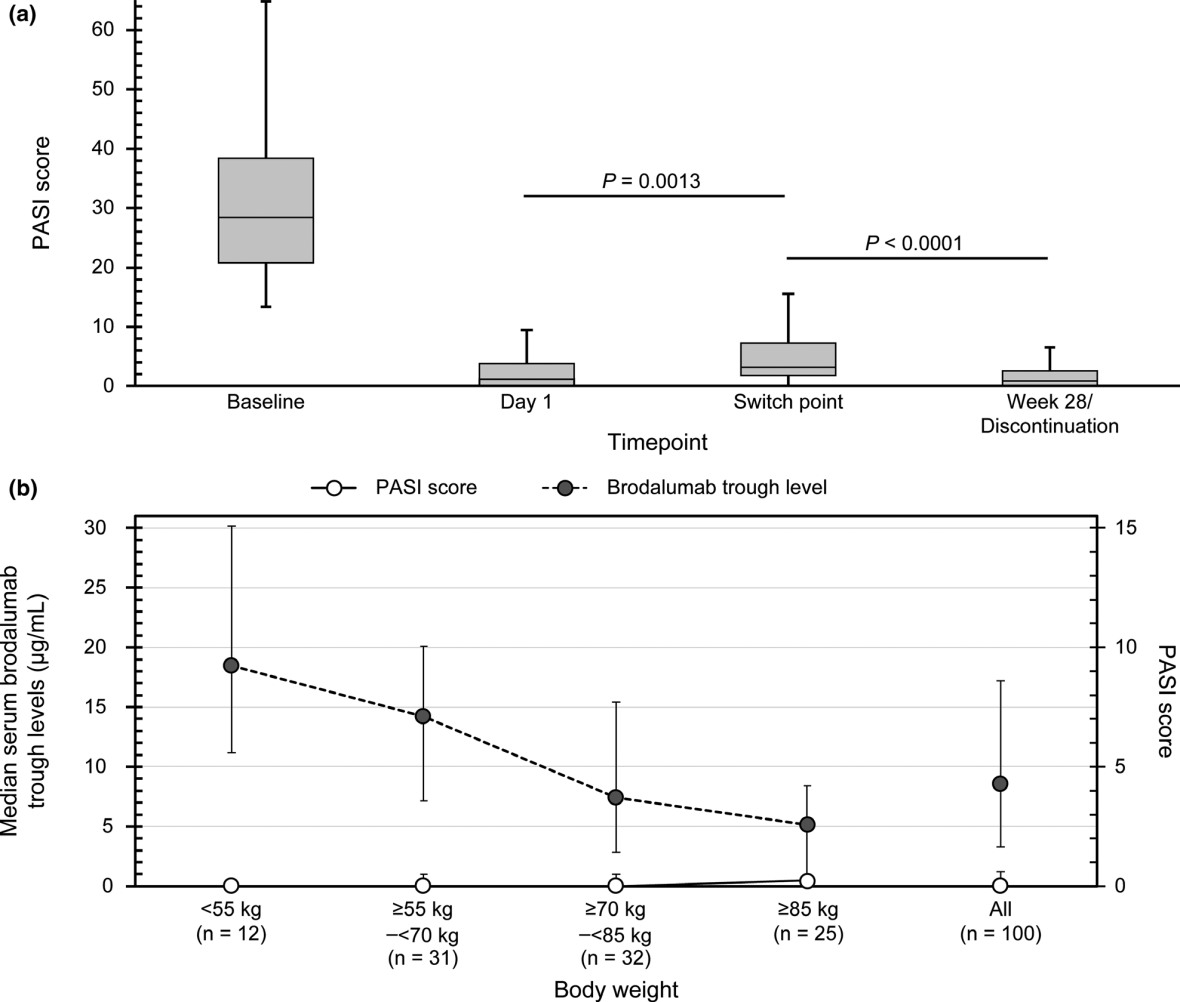
Efficacy with brodalumab 210 mg Q2W. (a) PASI score in patients who switched to 210 mg Q2W by Week 28. (b) Serum brodalumab levels and PASI score by body weight in patients on 210 mg Q2W at EOS. EOS, end of study; Q2W, every 2 weeks; Q4W, every 4 weeks; PASI, Psoriasis Area and Severity Index. In Fig. 3a, the horizontal lines in the boxes represent the following values: top, quartile 3; middle, median; bottom, quartile 1. The vertical lines on each box represent: top: quartile 3 + 1.5 × interquartile range; bottom: minimum values.

PASI score significantly (*P* < 0.001) decreased in 55 patients on 210 mg Q2W at Week 28 (Fig. 1) after dosage switch (Fig. 3b).

### Serum brodalumab levels, body weight, and PASI score with 210 mg Q2W at EOS

In patients on 210 mg Q2W at EOS, serum trough levels decreased with increasing body weight (Fig. 3c). Although the PASI score did not change in relation to body weight (Fig. 3c), the proportion of patients with PASI score ≤2 decreased with increasing body weight (<55 kg, 100%; ≥55–<70 kg, 96.8%; ≥70–<85 kg, 90.6%; ≥85 kg, 72.0%; data not shown). A multiple linear regression model adjusted by serum brodalumab levels and body weight showed a significant association between body weight and PASI score (95% confidence interval, 0.032–0.123; *P* = 0.001; Table 1).

**Table 1:** PASI score based on body weight and serum brodalumab levels

Variable	Parameter estimate	SE	95% CI	*t* Value	*P*‐value	Adjusted *R*‐squared
Intercept	−4.704	2.023	−8.720 to −0.688	−2.325	0.022	0.128
Body weight	0.078	0.023	0.032 to 0.123	3.401	0.001	
Brodalumab trough levels	−0.005	0.039	−0.084 to 0.073	−0.138	0.890	

Abbreviations: CI, confidence interval; PASI, Psoriasis Area and Severity Index; SE, standard error.

### Efficacy in patients on 140 mg Q4W

**Figure 4:**
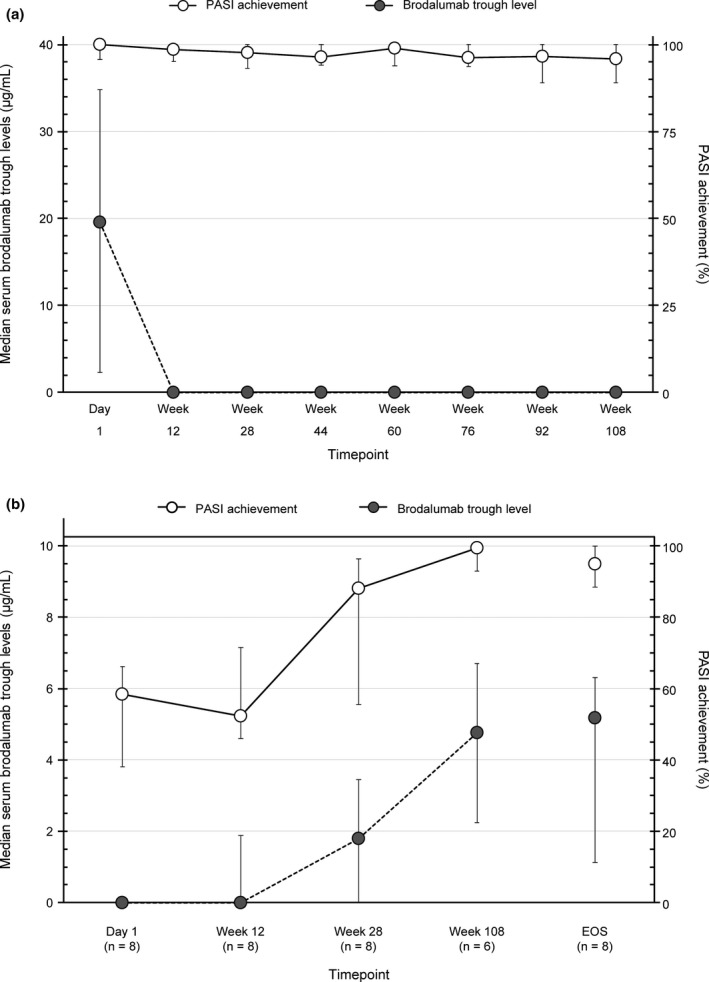
Efficacy in other subgroups. (a) Serum brodalumab levels and PASI response with 140 mg Q4W from Day 1 to Week 108. (b) Serum brodalumab levels and PASI response in responders to brodalumab 210 mg Q2W at EOS, but no response on Day 1. EOS, end of study; PASI, Psoriasis Area and Severity Index; Q2W, every 2 weeks; Q4W, every 4 weeks.

In eight patients who maintained the 140 mg Q4W dose from Day 1 to Week 108, a high PASI response was maintained despite absent serum trough levels starting Week 12 (Fig. 4a). Body weight of these eight patients over the study is shown in Fig. S2.

### Non‐responders among patients on 210 mg Q2W at EOS

Of the 100 patients on 210 mg Q2W at EOS, eight (8.0%) who were non‐responders (did not achieve PASI 75) on Day 1 achieved PASI ≥ 75 by EOS; PASI response increased in proportion with an increase in serum trough levels (Fig. 4b). Of 4/100 non‐responders at EOS (Table 2), three were non‐responders and one had PASI 100 on Day 1. All four patients had a high body weight (>87 kg) and serum trough levels of 0 from Day 1 to EOS.

**Table 2:** PASI achievement rate, serum brodalumab levels, and body weight in non‐responders on 210 mg Q2W at EOS

	EOS (week)	PASI achievement rate from baseline (%)		Brodalumab trough levels (μg/mL)		Body weight from Day 1 to EOS (kg)
		Day 1	EOS	Day 1	EOS	Minimum
Patient 1	124	−55.6	−32.1	0.00	0.00	119.0
Patient 2	20	34.8	−43.7	0.00	0.00	88.0
Patient 3	44	39.5	59.3	0.00	0.00	133.7
Patient 4	100	100.0	59.4	0.05	0.00	87.8

Abbreviations: EOS, end of study; PASI, Psoriasis Area and Severity Index; Q2W, every 2 weeks.

## Discussion

In this long‐term study in Japanese patients with plaque psoriasis, the association of efficacy with serum brodalumab levels was evaluated by changing the treatment dosage at the physician’s discretion and/or based upon treatment response until approval of brodalumab in Japan, following which the dose was adjusted and maintained in all patients.

Overall, serum brodalumab levels significantly increased with increasing dosage, as was previously observed in healthy volunteers and patients with psoriasis receiving brodalumab.^13^ Upon switching to 210 mg Q2W in patients receiving 140 mg Q4W on Day 1, improvement in efficacy was observed as a significant decrease in PASI score, suggesting that efficacy can be restored by switching to 210 mg Q2W, resulting likely from optimizing serum brodalumab levels. Taken together, these results emphasize the importance of using brodalumab at the approved dose to achieve maximum efficacy. The absence of a PASI score of 0 in all patients with measurable serum brodalumab levels is suggestive of the involvement of pathways other than those mediated by IL‐17receptor, such as IL‐36 agonists, IL‐36 receptors, and IL‐38 antagonists.^21^, ^22^

Previous studies in patients with psoriasis have demonstrated increased clearance^15^ and low serum brodalumab levels in patients with high body weight.^12^, ^17^ In the current study, serum brodalumab levels decreased with increasing body weight in patients on 210 mg Q2W at EOS. Further, the four non‐responders on 210 mg Q2W maintained a high body weight throughout the study and had serum brodalumab levels below LLOQ, suggesting that body weight adversely impacts serum brodalumab levels in patients with plaque psoriasis. PASI scores also improved with an increase in serum brodalumab levels, but the difference between score categories (except for 0 vs. >2 at Week 28) was not statistically significant.

In the current study, patients with body weight ≥85 kg tended to have a slightly higher PASI score; however, PASI scores were not affected by body weight, similar to the results of a *post hoc* analysis of the AMAGINE‐2 and −3 trials of brodalumab in which PASI 100 rates were comparable between non‐obese (body mass index [BMI] <30 kg/m^2^) and obese (BMI ≥ 30 kg/m^2^) patients with psoriasis.^23^ A larger variation in serum brodalumab levels was observed at PASI scores ≥2, with patients having PASI score >5 showing serum trough levels below LLOQ. Notably, the proportion of patients with a PASI score ≤2 decreased with increasing body weight. An earlier meta‐analysis of clinical studies in obese/overweight patients with psoriasis revealed a reduction in PASI score by ~2.5 and an increase in PASI 75 response rate among those who underwent a weight loss intervention.^24^ The current results suggest that body weight is an independent predictor of PASI scores regardless of serum brodalumab levels. Moreover, current recommendations suggest management of obesity in patients with psoriasis.^25^ Thus, it is imperative that body weight is routinely monitored and managed in clinical practice in patients with psoriasis, but dosage variations based on body weight should be implemented carefully.

Measurable serum brodalumab levels were only detected in responders. A previous analysis of the current/^18^ preceding^26^ study data demonstrated an absence of neutralizing anti‐brodalumab antibodies. Therefore, the lack of efficacy in non‐responders could be related to poor adherence, given that adherence to biologics is reported to be suboptimal.^27^ Moreover, self‐administration of brodalumab was permitted from Day 1. Thus, treatment adherence should be routinely monitored in clinical practice. Interestingly, PASI response was maintained in eight patients on 140 mg Q4W up to 108 weeks who had no measurable serum brodalumab levels. The presence of a PASI response could be related to reversal of selective gene expression to non‐lesional levels in the affected skin^28^, ^29^ or to receptor occupancy.^30^ However, receptor occupancy is reportedly at a maximum at a serum brodalumab levels of >~1 μg/mL. Thus, the efficacy of brodalumab in patients with absent serum drug levels needs to be further explored.

These results should be interpreted with caution, given that this was a *post hoc* analysis involving a small sample and was therefore not powered to evaluate all parameters. Moreover, there was no active comparator or control. Further, the serum brodalumab levels were not analyzed using an area under the concentration‐time curve, and efficacy was assessed only at certain trough levels with varying doses. Additionally, serum levels are not reflective of brodalumab levels in the rash or receptor occupancy, which were not measured in this study. Finally, body weight, and not change in body weight/BMI/body fat percentage, was assessed at each timepoint, which may have impacted the assessment of the association between body weight and efficacy.

In conclusion, our results suggest that in patients with plaque psoriasis, efficacy of brodalumab may depend upon sustained serum trough levels and can be restored with the use of the approved dose. Further, good treatment adherence and body weight management can help in maximizing the efficacy of brodalumab in daily clinical practice.

## Conflicts of Interest

Y. Y. reports personal fees and non‐financial support from Kyowa Kirin during conduct of the study; and grants from Ministry of Education, Culture, Sports, Science and Technology, and Japan Agency for Medical Research and Development (AMED); personal fees from AbbVie, Celgene, Janssen Pharmaceutical, Novartis Pharma, Boehringer Ingelheim, LEO Pharma, and Sanofi; and grants and personal fees from Kyowa Kirin, Maruho, Torii Pharmaceutical, Mitsubishi Tanabe Pharma, and Eli Lilly Japan, outside the submitted work. Y. K. and H. O. are employees of Kyowa Kirin. H. K. is an employee of and holds stock of Kyowa Kirin. H. N. reports personal fees from Kyowa Kirin during conduct of the study; and personal fees as consulting fees and/or speaker honoraria from AbbVie, Eisai, Eli Lilly Japan, Janssen Pharmaceutical, Japan Tobacco, Kyowa Kirin, LEO Pharma, Maruho, Novartis Pharma, Torii Pharmaceutical, and UCB Japan, outside the submitted work.

## Supporting information


**Figure S1**. Serum brodalumab levels in patients with PASI score >2 at Week 28 and Week 108.

**Figure S2**. Body weight in patients on 140 mg Q4W over the study.
