# Supplementary figures and images for: Relationship between serum trough levels and efficacy of brodalumab from a post hoc exploratory analysis of a Japanese study in patients with plaque psoriasis
Source: J Dermatol. 2020 Nov 8;48(3):324–33. doi: 10.1111/1346-8138.15690 (PMC7984373; doi:10.1111/1346-8138.15690)

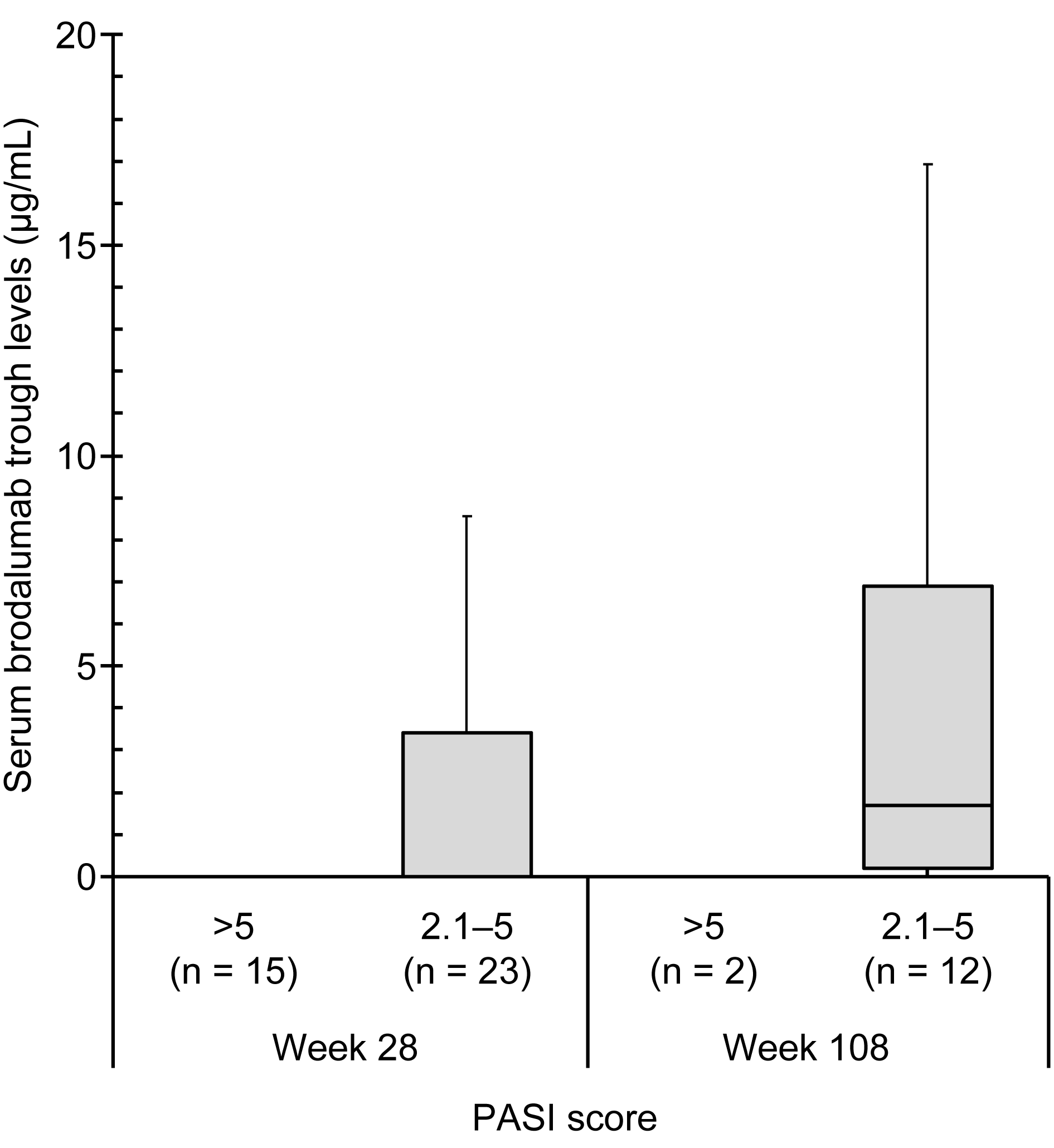

Supplement: Supplementary file 1 — Figure S1. Serum brodalumab levels in patients with PASI score >2 at Week 28 and Week 108. [file JDE-48-324-s001.tif]

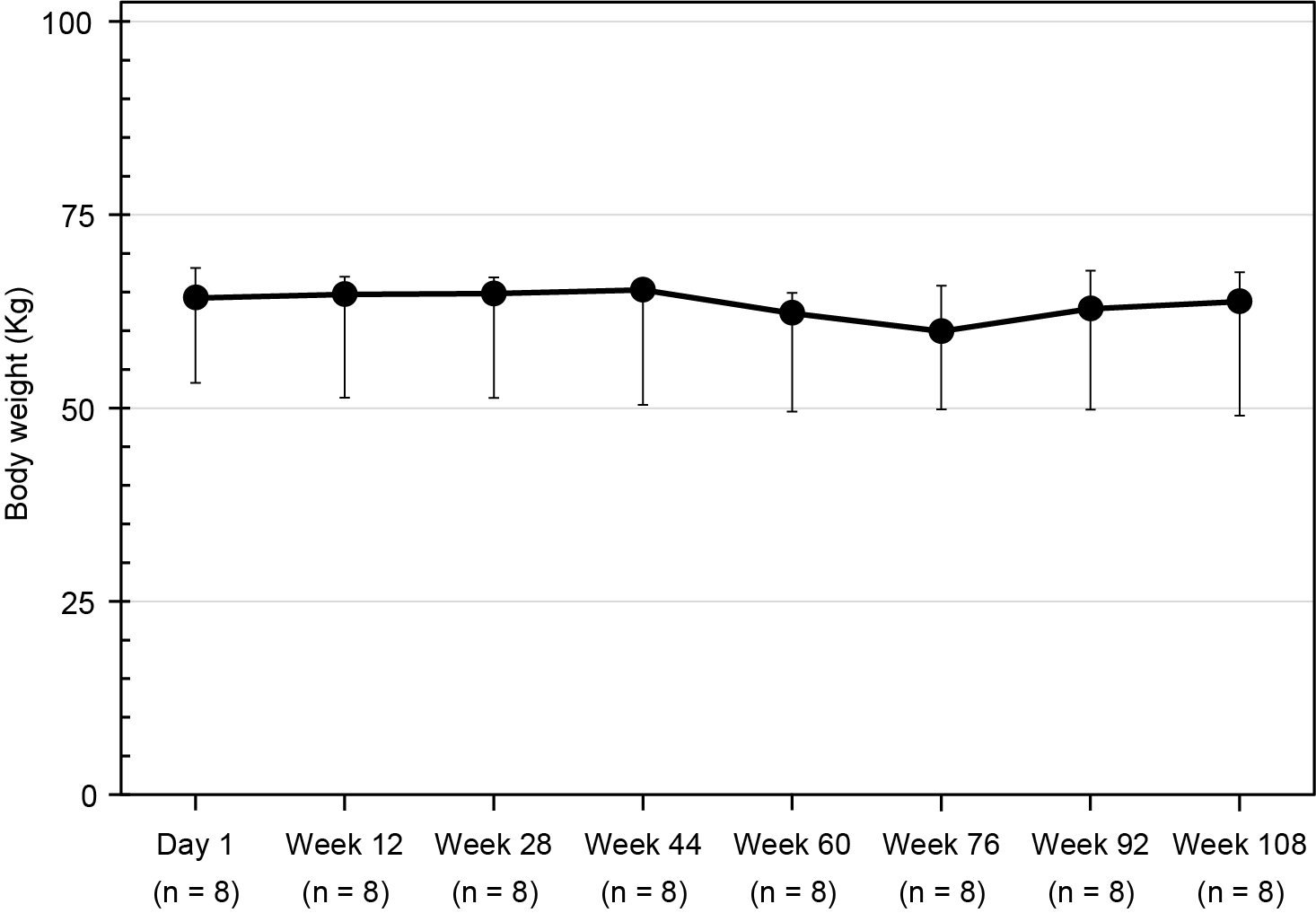

Supplement: Supplementary file 2 — Figure S2. Body weight in patients on 140 mg Q4W over the study. [file JDE-48-324-s002.tif]
